# Controlled pDNA Release in Gemini Cationic Lipoplexes by Femtosecond Laser Irradiation of Gold Nanostars

**DOI:** 10.3390/nano11061498

**Published:** 2021-06-05

**Authors:** Natalia Sánchez-Arribas, Pablo Díaz-Núñez, José Osío Barcina, Emilio Aicart, Elena Junquera, Andrés Guerrero-Martínez

**Affiliations:** 1 Departamento de Química Física, Facultad de Ciencias Químicas, Universidad Complutense de Madrid, 28040 Madrid, Spain; natsanch@ucm.es (N.S.-A.); aicart@quim.ucm.es (E.A.); 2 Instituto de Fusión Nuclear, “Guillermo Velarde”, Universidad Politécnica de Madrid, José Gutiérrez Abascal 2, 28006 Madrid, Spain; pablodn87@gmail.com; 3 Departamento de Química Orgánica, Facultad de Ciencias Químicas, Universidad Complutense de Madrid, 28040 Madrid, Spain; josio@quim.ucm.es

**Keywords:** lipoplexes, gene release-assisted, femtosecond pulse laser, gold nanostars

## Abstract

The design of nanovectors able to overcome biological barriers is one of the main challenges in biomedicine. Gemini cationic lipids are considered potential candidates for gene therapy due to their high biocompatibility and capacity to condense nucleic acids safely in the form of lipoplexes. However, this approach presents difficulties regarding genetic unpacking and, therefore, control over this process becomes crucial to ensure successful transfection. In this work, gemini cationic lipoplexes were prepared in the presence of plasmonic gold nanostars (AuNSs) to afford a nanovector that efficiently releases plasmid DNA (pDNA) upon irradiation with near-infrared femtosecond laser pulses. A critical AuNSs concentration of 50 pM and optimized laser power density of 400 mW led to successful pDNA release, whose efficiency could be further improved by increasing the irradiation time. Agarose gel electrophoresis was used to confirm pDNA release. UV-Vis-NIR spectroscopy and transmission electron microscopy studies were performed to monitor changes in the morphology of the AuNSs and lipoplexes after irradiation. From a physicochemical point of view, this study demonstrates that the use of AuNSs combined with gemini cationic lipoplexes allows control over pDNA release under ultrafast laser irradiation.

## 1. Introduction

Lipoplexes are systems based on lipids or lipid mixtures able to compact nucleic acids (NAs), commonly used in gene therapy [1]. Their structural resemblance to biological membranes confers these lipids with high biocompatibility, making them potential candidates to replace traditional viral vectors, which exhibit higher transfection efficiency but may trigger non-desired immune responses [2,3]. Cationic lipids (CLs) are particularly attractive for their ability to interact electrostatically with NAs. The positively charged lipoplexes thus formed are able to cross the negatively charged cellular membrane typically by endocytosis [4,5]. Gemini cationic lipids (GCLs) are probably the most studied CL family due to the high variability in their chemical structure, not only at the hydrophobic tails, but also at the cationic heads and spacers. GCL-type nanovectors have demonstrated their ability to successfully transfect NAs in different cell lines [6,7,8,9]. They have been especially used to transfect plasmid DNA (pDNA) with remarkable success when combined with a helper lipid [10,11,12], which usually promote cellular uptake as well as facilitate endosomal membrane rupture once inside the cell [11,13]. 

However, on many occasions, lipoplexes present low therapeutic efficiencies due to their inability to overcome the different biological barriers that separate them from the final target [4,14]. Therefore, the specific destabilization of the endosomes is one of the crucial factors of the release process. A low transfection efficiency is usually attributed to poor gene unpacking, whereby only a minor fraction of NAs is able to exit the endocytic vehicle [15]. Endosomal escape has to be a fast but safe process to avoid NA degradation and, thus, control over it becomes critical. Many studies have focused on nanovectors that respond to internal (i.e., changes in pH) or external (i.e., temperature or electric pulses) stimuli for release or transfection [16,17]. 

The localized surface plasmon resonances (LSPRs) of gold nanoparticles (AuNPs) render them with strong absorption efficiency and attractive photothermal properties that can be activated by external laser excitation [18]. As examples of biomedical applications, the irradiation of AuNPs with lasers induces the release of controlled quantities of antitumor drugs into cancer cells [19], or kills cancer cells in the case of plasmonic photothermal therapies (PPTT) [20]. In particular, plasmonic gold nanostars (AuNSs) are especially appealing because they exhibit broad LSPR bands, displaying very high electromagnetic fields localized at the tips, much larger than those displayed by other morphologies such as gold nanospheres or nanorods [21]. Additionally, the LSPR of AuNSs can be tuned to the near-infrared (NIR) region as a function of the width, length and number of spikes [22]. The NIR region is interesting from a biomedical point of view due to the well-known first (650–950 nm) and second (1000–1350 nm) biological windows, where the blood and water within organisms present low NIR absorption and scattering [23,24,25]. As an example, we have shown the use of AuNSs with an LSPR band centered at 800 nm for efficient PPTT on melanoma cells by femtosecond laser irradiation [26].

The use of femtosecond pulse lasers to irradiate AuNPs and promote photothermal processes is especially suited because the fast energy deposition rate leads to a high electronic temperature, which is transferred to the nanocrystal lattice via electron-phonon relaxation processes, heating the nanoparticles above the melting temperature of bulk gold [27]. On the other hand, in nanosecond pulse laser excitation, the absorption of photons continues when the relaxation processes have already started and the lattice is still hot, yielding an increase of the lattice energy that usually produces uncontrolled heating and undesired fragmentation of the nanocrystals [27].

**Scheme 1:**
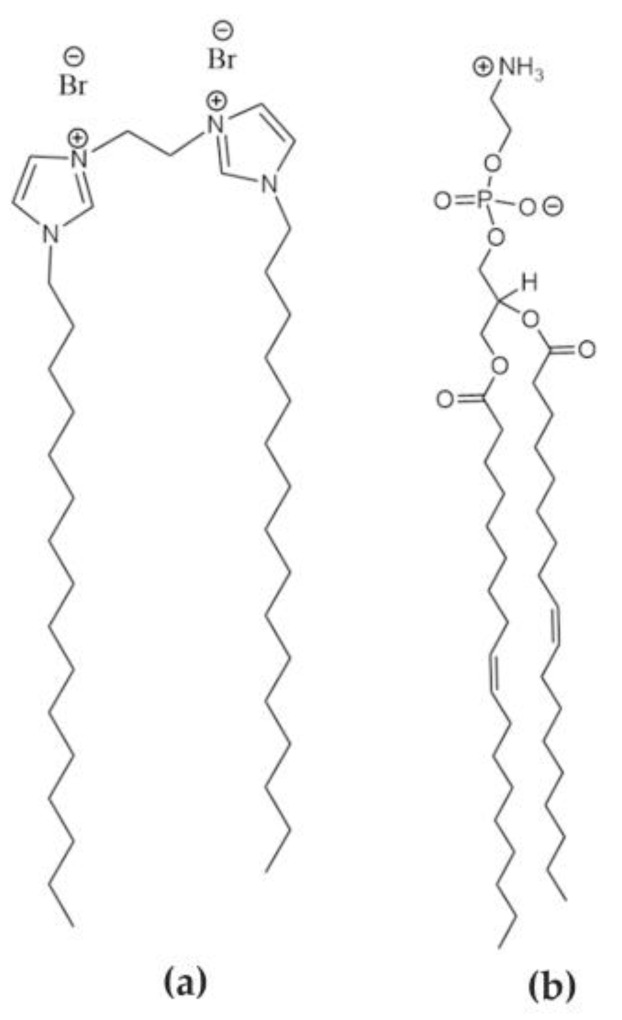
Molecular structure of the gemini cationic lipid IGCL (**a**) and zwitterionic lipid DOPE (**b**).

The aim of this investigation was to evaluate the controlled release of pDNA in gemini cationic lipoplex systems with the assistance of AuNSs upon irradiation with a femtosecond laser (800 nm, Ti:sapphire, 90 fs laser pulses, 1 kHz). AuNSs were synthesized and characterized by UV-Vis-NIR spectroscopy and transmission electron microscopy (TEM). The liposomes were constructed from gemini cationic lipid 1,2-bis(hexadecyl imidazolium) dialkane (IGCL) and helper zwitterionic lipid 1,2-dioleoyl-*sn*-glycero-3-phosphatidylethanolamine (DOPE) (Scheme 1), and the formation of the corresponding lipoplexes upon addition of pDNA were performed in the presence of AuNSs. Such lipid mixture was chosen due to the high ability of the resulting lipoplexes to compact pDNA [10]. The liposome-AuNS and lipoplex-AuNS systems were characterized and optimized by ζ–potential measurements, UV-Vis-NIR spectroscopy and/or TEM at different nanoparticle concentrations and femtosecond laser irradiation conditions. Agarose gel electrophoresis was used to analyze and quantify the pDNA release upon irradiation. 

## 2. Materials and Methods

### 2.1. Materials

The reagents used for AuNS synthesis (sodium citrate, gold(III) chloride trihydrate, hydrochloric acid, silver nitrate and ascorbic acid) and functionalization (poly(ethylene glycol) methyl ether thiol, 6 kDa) were purchased from Sigma-Aldrich (St. Louis, MO, USA) or Scharlab S.L. (Barcelona, Spain). The synthesis of IGCL has been previously reported [10], and zwitterionic lipid DOPE was supplied by Avanti Polar Lipids, Inc. (Alabaster, AL, USA). Both molecular structures are shown in Scheme 1. pEGFP-C3 plasmid DNA (4700 bp) (pDNA) was extracted from competent *Escherichia coli* bacteria using a GenElute HP Select Plasmid Gigaprep kit (Sigma-Aldrich).

### 2.2. Optical Characterization

All spectra were recorded at room temperature on a BioTek Instruments Uvikon XL UV-Vis-NIR spectrophotometer, using quartz cuvettes with a 1 mm transmission path length.

### 2.3. AuNSs Synthesis

AuNSs were prepared by a modified seed-mediated growth method [28] in water. Firstly, a seed solution was prepared by adding 5 mL of citrate solution (1%) to 95 mL of a boiling HAuCl_4_ (0.45 mM) solution under vigorous agitation. The gold seeds were citrate-stabilized after boiling for 5 min (red colloid). The seed solution was cooled down to room temperature. Then, 750 µL of seeds were added under gentle stirring to 50 mL of a HAuCl_4_ (0.25 mM) solution containing 50 μL of HCl (1.0 M). Next, 500 μL of AgNO_3_ (3 mM) and 250 μL of ascorbic acid (100 mM) were added simultaneously. Finally, 500 μL of PEG-SH (0.2 mM) was incorporated and mixed for 30 min to stabilize the AuNSs (blue colloid). AuNSs were washed by centrifugation at 1190× *g* and 10 °C for 30 min. Then, the final AuNS colloids were redispersed in *N*-(2-hydroxyethyl)piperazine-*N’*-ethanesulfonic acid (HEPES)-buffered medium at physiological conditions (40 mM, pH = 7.4).

### 2.4. Preparation of Lipoplex-AuNS System

Appropriate amounts of IGCL and DOPE solutions in chloroform were mixed to obtain the optimal IGCL molar composition (α = 0.2) [10]. The solvent was evaporated under high vacuum to form a dry lipid film. Then, the film was hydrated and homogenized with the AuNSs colloidal solution at different nanoparticle concentrations by alternating vortex/sonication cycles. The liposome-AuNS system thus obtained was incubated with pDNA at room temperature for 30 min, affording the lipoplex-AuNS system. The pDNA concentration in this system was set at 0.05 mg/mL for ζ–potential measurements and at 0.02 mg/mL for the femtosecond laser irradiation experiments.

The lipoplex-AuNS system was formed at selected mass ratios of lipid mixture and pDNA (m_L_/m_pDNA_), following Equation (1):

(1) $$m_{L}{/m}_{\mathrm{pDNA}}={(m}_{\mathrm{IGCL}}{+m}_{\mathrm{DOPE}}{)/m}_{\mathrm{pDNA}}$$

being m_L_, m_IGCL_, m_DOPE_ and m_pDNA_ the mass of the total mixed lipid, IGCL, DOPE, and pDNA, respectively.

The lipoplex-AuNS system was prepared at the selected effective charge ratio given by Equation (2):

(2) $$\rho_{\mathrm{eff}}=\frac{n^{+}}{n^{-}}\;=\frac{q_{\mathrm{eff},\mathrm{IGCL}}^{+}{(m}_{\mathrm{IGCL}}{/M}_{\mathrm{GCL}})}{q_{\mathrm{eff},\mathrm{pDNA}}^{-}{(m}_{\mathrm{pDNA}}/{\bar{M}}_{\mathrm{pDNA}/\mathrm{bp}})}$$

where n^+^, n^−^, $q_{\mathrm{eff},\mathrm{IGCL}}^{+}$, $q_{\mathrm{eff},\mathrm{pDNA}}^{-}$, M_IGCL_ and M_pDNA_ are the number of moles of positive (IGCL) and negative (pDNA) charges, effective charge of IGCL and pDNA per bp, and the molecular weight of IGCL and pDNA per bp, respectively.

### 2.5. ζ–Potential Measurements

The ζ–potential of the AuNSs and those of the liposomes and lipoplexes in the absence and presence of AuNSs (100 pM) were determined by electrophoretic mobility measurements using the phase analysis light scattering technique (Zeta PALS, Brookhaven Instruments Corp., Holtsville, NY, USA). The experimental conditions selected were a temperature of 25 °C, dispersant refractive index of 1.33, viscosity of 0.9 cP, and dispersant dielectric constant of 78.5. Each value of ζ–potential is the average of 50 independent measurements, collected in a sigmoidal curve as a function of the mass ratio (m_L_/m_pDNA_) [29,30].

### 2.6. Femtosecond Laser Irradiation

The system included an amplified Ti:sapphire laser (Tsunami, Spectra-Physics, Santa Clara, CA, USA) centered at 800 nm, with a pulse duration of 90 fs and repetition rate of 1 kHz. The laser power density was controlled by a variable neutral density filter, with a laser spot diameter of 5 mm. The lipoplex-AuNS system at selected nanoparticle concentrations (from 20 to 200 pM) was irradiated in a 96-well plate at different times (from 5 to 60 min) and femtosecond laser power densities (from 50 to 700 mW).

### 2.7. Agarose Gel Electrophoresis 

The release of pDNA after femtosecond laser irradiation of the lipoplex-AuNS system was determined by agarose gel electrophoresis. Free pDNA (used as the control) and the lipoplex-AuNS samples (at different conditions of irradiation) were loaded in a 0.8% (*w/v*) agarose gel in 1× TAE buffer with 0.7 µL of GelRed probe. Electrophoresis was run at 100 mV and 400 mA for 1 h. The agarose gels were visualized using a Gel Doc XR instrument (Bio-Rad, Hercules, CA, USA) running the Quantity One software, which allowed quantification of the trace pDNA bands (Int*mm). Probe emission was excited at 302–312 nm and recorded at 600 nm.

### 2.8. Transmission Electron Microscopy

TEM micrographs were recorded on a JEOL JEM 1400 transmission electron microscope operating at an acceleration voltage of 120 kV. In the stained TEM images, both non-irradiated and irradiated systems were fixed with a solution containing 4% paraformaldehyde and 2.5% glutaraldehyde in HEPES for 2 h at 4 °C. After three washes (at 10,000 rpm, 10 °C, 15 min), the samples were stained with 1% osmium tetroxide in HEPES solution for 1 h at room temperature. The samples were further washed three times (at 10,000 rpm, 10 °C, 15 min), gradually dehydrated in solutions of acetone, and finally embedded in Epon. For TEM observation, the samples were previously cut by ultramicrotomy.

## 3. Results and Discussion

### 3.1. Synthesis and Characterization of AuNSs

**Figure 1:**
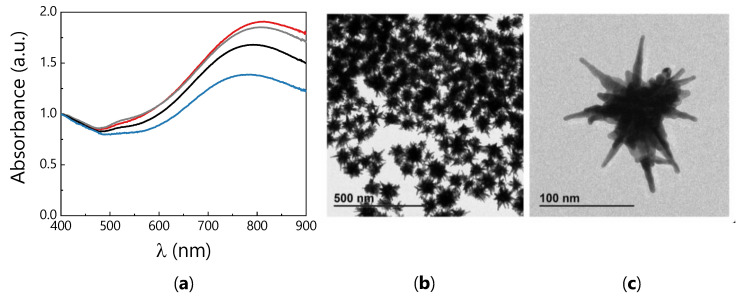
(**a**) Normalized UV-Vis-NIR spectra of the AuNSs with LSPR bands centered at 812 nm in water (red line) and 805 nm in HEPES-buffered medium (gray line), and of the liposome-AuNS and lipoplex-AuNS systems with the LSPR band centered at 785 (black line) and 780 nm (blue line) and (**b**,**c**) TEM micrographs of AuNSs at low and high magnifications.

Plasmonic AuNSs were synthesized following the colloidal seed-mediated growth method (Materials and Methods section). Citrate seeds were used to grow AuNSs, later functionalized with PEG-SH. Polyethylene glycol was used to confer high stability and biocompatibility to the nanoparticles, avoiding their aggregation at physiological ionic strengths [31]. The AuNSs exhibited a broad LSPR band centered at 812 nm in water (Figure 1a), which can be assigned to a dipolar resonance localized at the tips [21]. A slight blue shift of the LSPR band to 805 nm was observed in HEPES-buffered medium, as a consequence of the changes in the chemical environment [32]. AuNSs with cores of 55.0 ± 10.0 nm and spikes of 50 ± 25 nm were visualized by TEM (Figure 1b,c).

### 3.2. Formation and Characterization of the Lipoplex-AuNS System

**Figure 2:**
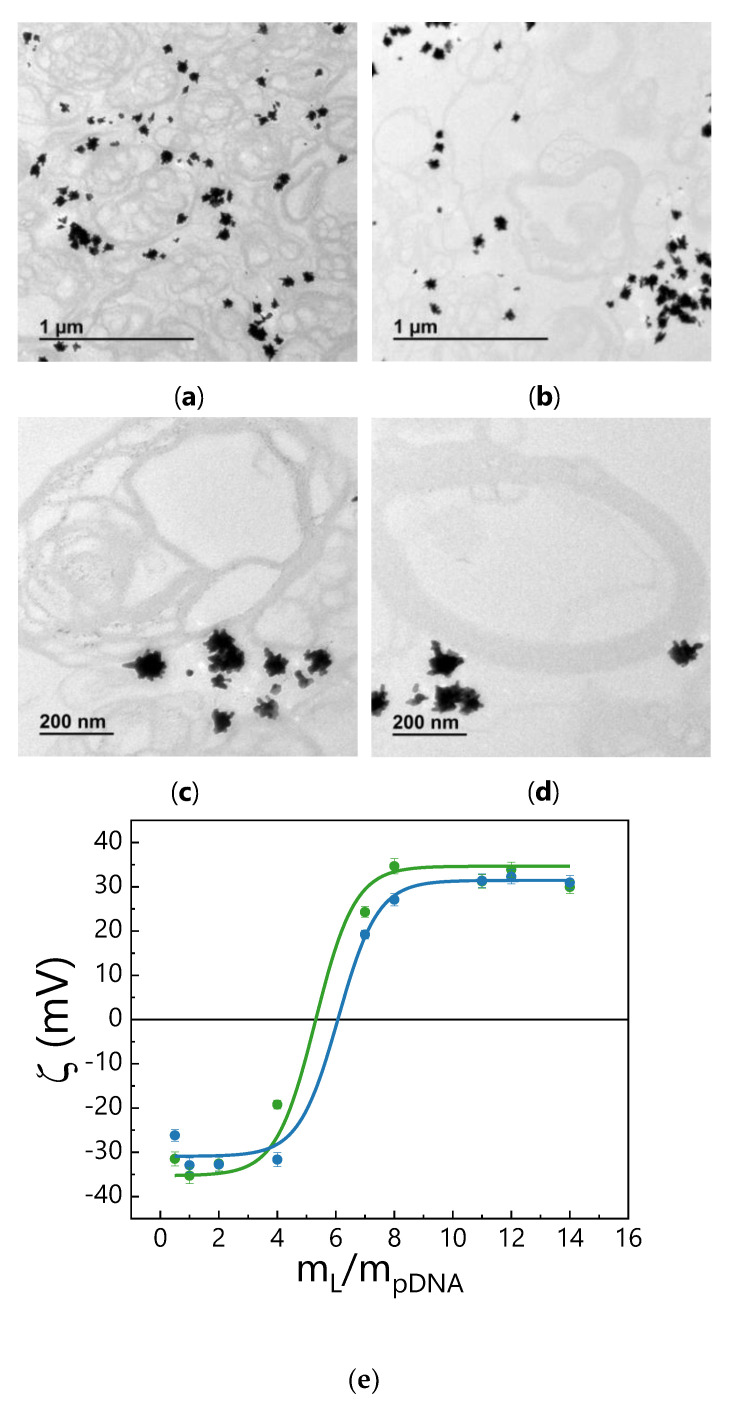
Stained TEM micrographs of the liposome-AuNS (**a**,**c**) and lipoplex-AuNS (**b**,**d**) systems and (**e**) ζ–potential versus m_L_/m_pDNA_ of the lipoplex (green points) and lipoplex-AuNS (blue points) systems.

The dry film formed by IGCL and DOPE was hydrated with the AuNSs colloid solution to form the liposome-AuNS and lipoplex-AuNS systems upon addition of pDNA (Materials and Methods section). The LSPR bands of the nanoparticles in the liposome-AuNS and lipoplex-AuNS systems displayed a blue shift to 785 nm and 780 nm in HEPES-buffered medium (Figure 1a), respectively, due to changes in the chemical environment around the AuNSs. The decrease in intensity of the normalized LSPR band in both systems was attributed to an increase in the spectral background resulting from incorporation of the lipid mixture and pDNA. As shown by the stained TEM micrographs in Figure 2a,c, the AuNSs were not in direct contact with the liposomes, but coexisted in the lipid mixture by confinement in the inter-liposome space. Figure 2b,d shows that the presence of AuNSs did not influence the formation of lipoplexes, for which a typical lamellar pattern was still observed and where the nanoparticles remained at the inter-lipoplex region. Therefore, in the presence of these nanoparticles, the stability of the IGCL/DOPE liposomes and lipoplexes is retained, and also the plasmonic properties of the AuNSs, ensuring thus the possibility of irradiation with femtosecond lasers at 800 nm.

The ζ–potential of the lipoplexes was studied in the absence and presence of AuNSs. Figure 2e shows the typical sigmoidal profile of ζ–potential measurements vs. m_L_/m_pDNA_ (Equation (1)) in the lipoplex (green points) and lipoplex-AuNS (blue points) systems. The ζ–potential values were similar, showing that the presence of AuNSs did not hinder the formation and stability of lipoplexes (Figure 2b,d), in good agreement with the analogous ζ–potential values obtained for the liposome system in the absence (32 ± 2 mV) and presence (36 ± 2 mV) of AuNSs. Such coexistence was further supported by the null value of the ζ–potential obtained in the case of free AuNSs (0 ± 1 mV). The Boltzmann-type fit provided an electroneutrality ratio (that is, the composition at which the net charge of lipoplex changes from negative to positive) of (m_L_/m_pDNA_)_ϕ_ = 5.3 ± 0.3 for the lipoplex and of 6.0 ± 0.3 for the lipoplex-AuNS system. Following a protocol described elsewhere [29,30], these values were used to obtain $q_{\mathrm{eff},\mathrm{pDNA}/\mathrm{bp}}^{-}$ in the absence and presence of AuNSs, which indicated analogous effective pDNA charges per bp in both systems (−1.6 ± 0.1 and −1.8 ± 0.1, respectively). Finally, the values of $q_{\mathrm{eff},\mathrm{pDNA}/\mathrm{bp}}^{-}$ in the presence of AuNSs and $q_{\mathrm{eff},\mathrm{GCL}}^{+}$ (+1.7 ± 0.1) [10] were used to determine the effective charge ratio (Equation (2)) of the lipoplex-AuNS system (ρ_eff_ = 5), at which the lipoplexes are positively charged at moderate IGCL concentrations.

### 3.3. Femtosecond Laser Irradiation of the Lipoplex-AuNS System

**Figure 3:**
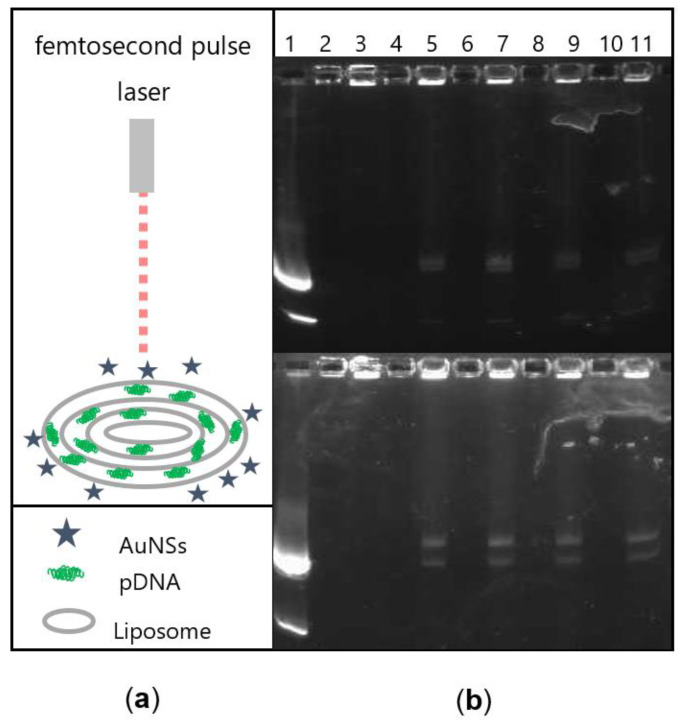
(**a**) Scheme of irradiation and (**b**) Agarose gel electrophoresis of lipoplex-AuNS systems under non-irradiated (lanes 2, 4, 6, 8 and 10) and irradiated (lanes 3, 5, 7, 9 and 11) conditions, at 400 mW (top panel) and 700 mW (bottom panel) for 5 min. The lipoplex-AuNS systems were prepared at different AuNS concentrations: 20 pM (lanes 2–3), 50 pM (lanes 4–5), 100 pM (lanes 6–7), 150 pM (lanes 8–9) and 200 pM (lanes 10–11). Free pDNA was used as the control (lane 1).

Among the plasmonic properties of AuNSs, they display high plasmonic efficiency at 800 nm (Figure 1a). By controlled irradiation with femtosecond laser pulses at such wavelength, the thermal heating thus generated can be exploited to disrupt the lipoplex structure, thereby inducing the release of pDNA from the lipoplex-AuNS system (see scheme in Figure 3a). 

Agarose gel electrophoresis was used to monitor the pDNA release after irradiation (Materials and Methods section). Figure 3b shows the influence of the AuNS concentration and laser power density on the pDNA release. The lipoplex-AuNS system was prepared at different nanoparticle concentrations at which AuNSs are not cytotoxic [26] (20, 50, 100, 150 and 200 pM). After irradiation at the optimal laser power density of 400 mW (Figure 3b, top panel) for 5 min, the lipoplex-AuNS systems were loaded in lanes 3, 5, 7, 9 and 11, at increasing order of nanoparticle concentration. The corresponding non-irradiated lipoplex-AuNS systems were also loaded in lanes 2, 4, 6, 8 and 10, respectively. The fluorescent bands in lane 1 (free pDNA) are attributed to the coiled (upper band) and supercoiled (lower band) free pDNA conformations.

The lack of bands in lanes 2, 4, 6, 8 and 10 confirms the full compaction of pDNA by the lipid mixture in the presence of AuNSs. Similar results were obtained for the lipoplexes in the absence of AuNSs (see agarose gel electrophoresis image in Figure S1). No fluorescent bands were observed in lane 3 (20 pM AuNSs, after irradiation) (Figure 3b, top panel). From lane 5, the observation of bands indicates the release of pDNA in the irradiated systems. Therefore, a critical AuNSs concentration of 50 pM was concluded to be necessary to obtain enough thermal heating to trigger the release process. No significant increases in the fluorescence intensity of the bands were observed above this critical NP concentration. Additionally, equivalent lipoplex-AuNS systems irradiated at laser power densities below 400 mW for 5 min did not show significant pDNA release. In contrast, an increase of the laser power density to 700 mW resulted in more intense fluorescence (Figure 3b, bottom panel). Noticeably, the absence of new bands at both laser power densities indicated that no fragmentation of pDNA had occurred.

**Figure 4:**
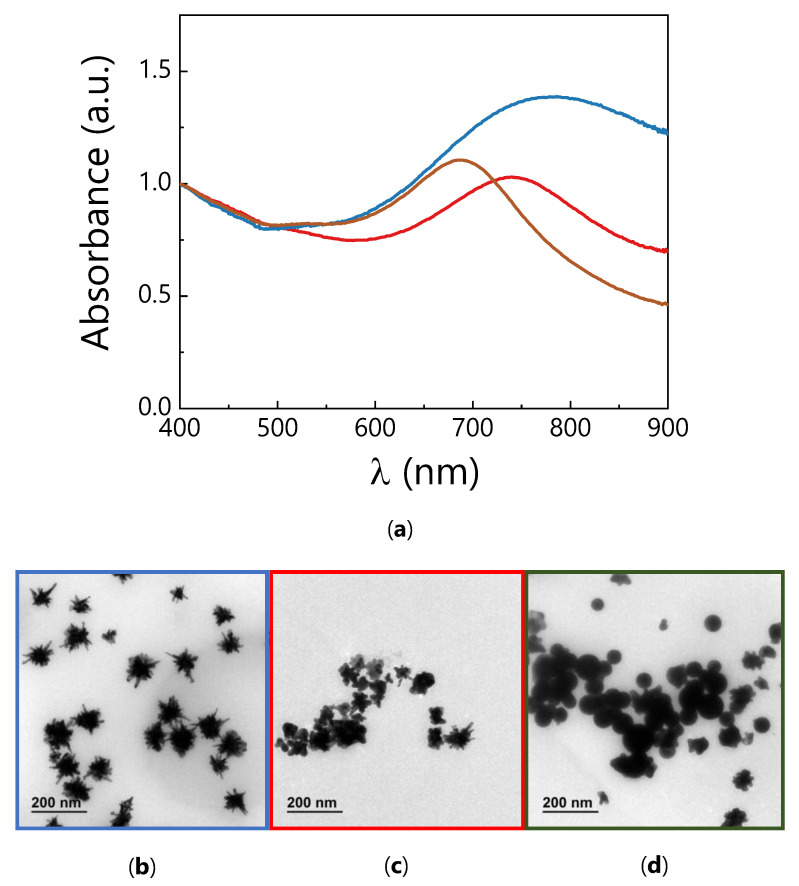
(**a**) Normalized UV-Vis-NIR spectra of the lipoplex-AuNS system (100 pM AuNSs) under non-irradiated (blue line) and irradiated conditions, at 400 mW (red line) and 700 mW (brown line) for 5 min. TEM micrographs of lipoplex-AuNS systems (100 pM AuNSs) under non-irradiated (**b**) and irradiated conditions at 400 mW (**c**) and 700 mW (**d**) for 5 min.

The effect of femtosecond laser irradiation on the lipoplex-AuNS system was studied by UV-Vis-NIR spectroscopy and TEM. Figure 4 shows an example of 100 pM AuNSs after irradiation. Under 400 and 700 mW for 5 min, noticeable changes in the LSPR band were observed, with blue shifts of 39 and 92 nm, respectively (Figure 4a). Analogous results were observed at different AuNS concentrations (Figure S2). These blue shifts indicate reshaping from star to spherical morphology with the increasing laser power density. The relatively long time of irradiation suggested the presence of an absorption inner filter effect, in which AuNSs that were firstly affected by the incident laser pulses absorbed more energy, and AuNSs behind needed more time to absorb enough energy to be sufficiently heated; this process is governed by the Brownian motion of the nanoparticles within the colloid. No significant changes in the LSPR band of AuNSs were appreciated at laser power densities below 400 mW (Figure S3). These results were corroborated by TEM imaging, whereby the number and length of spikes of AuNSs were seen to decrease with the increasing laser power density until the final formation of nanospheres (Figure 4b–d). Similar conclusions were drawn from irradiation experiments of the lipoplex-AuNS systems at different nanoparticle concentrations (Figures S4 and S5).

**Figure 5:**
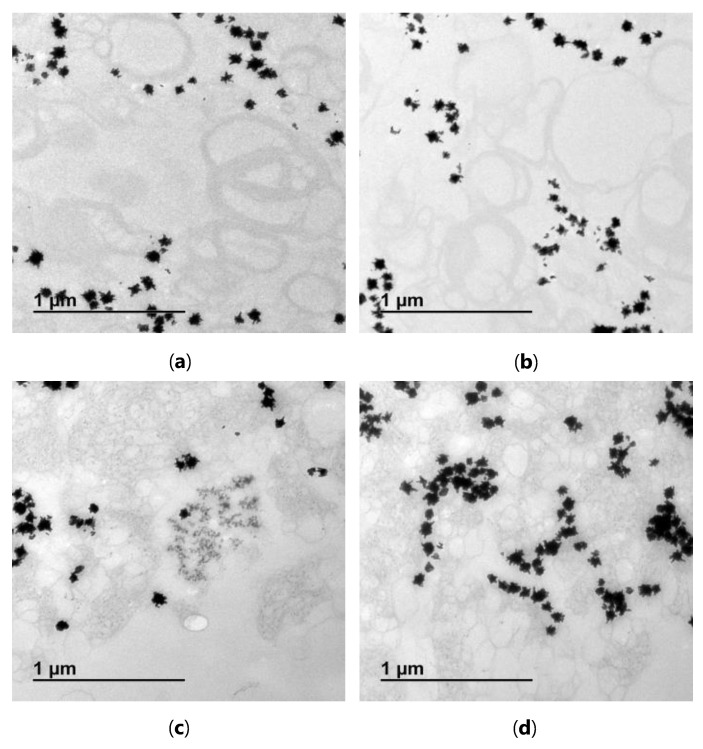
Stained TEM micrographs of the lipoplex-AuNS system (100 pM AuNSs) at irradiation conditions of 100 mW (**a**,**b**) and 400 mW (**c**,**d**) for 5 min.

The structure of the lipoplexes in the presence of nanoparticles (100 pM AuNSs) after femtosecond laser irradiation at 100 and 400 mW was next investigated by TEM. Figure 5a,b shows that the characteristic lipidic pattern (lamellar structure, Figure 2b,d) was not affected at 100 mW. Interestingly, no morphological modifications of the AuNSs were observed at that laser power density (Figure S3d). At 400 mW, however, the lipid bilayer structure could not be clearly identified (Figure 5c,d), suggesting the disruption of the lipoplexes by thermal heating, therefore triggering the pDNA release.

**Figure 6:**
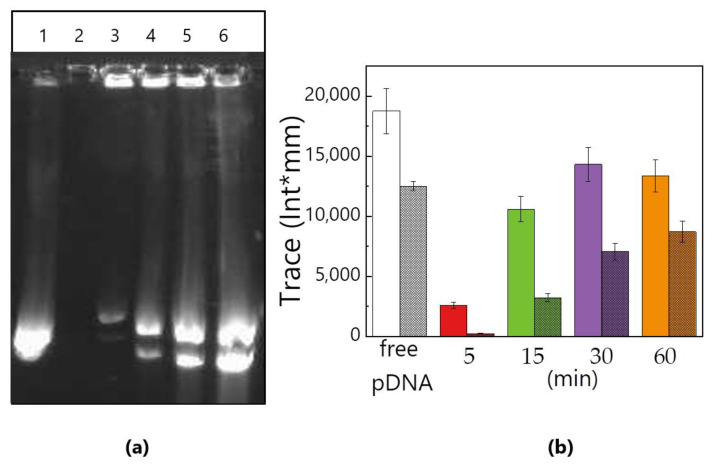
(**a**) Agarose gel electrophoresis of the lipoplex-AuNS system (100 pM AuNSs) irradiated at 400 mW at different times: 5 min (lane 3), 15 min (lane 4), 30 min (lane 5) and 60 min (lane 6). Lane 1: free pDNA as the control; lane 2: non-irradiated lipoplex-AuNS system and (**b**) Quantification of the pDNA release as a function of the time of irradiation (min). Traces (Int*mm) of the fluorescent bands of coiled (unstriped bars) and supercoiled (striped bars) pDNA conformations.

So as to optimize the pDNA release efficiency, the effect of the irradiation time (5, 15, 30 and 60 min) on the lipoplex-AuNS system (100 pM AuNSs) at the lowest effective laser power density (400 mW) was studied. Figure 6a shows the agarose gel for the electrophoresis experiment. Irradiated systems were loaded in lanes 3, 4, 5 and 6, at increasing order of irradiation time. The free pDNA control and the corresponding non-irradiated lipoplex-AuNS system were loaded in lanes 1 and 2, respectively. Figure 6b shows the quantification of the two fluorescent bands in each lane, corresponding to the coiled (unstriped bars) and supercoiled (striped bars) pDNA conformations. The intensity of the band fluorescence increased with the irradiation time and, with it, the efficiency of pDNA release. Note that the pDNA release achieved efficiency values up to 75% at times longer than 15 min. Again, no additional fragments of pDNA were observed with the increasing irradiation time. 

Analysis of the UV-Vis-NIR spectra of these lipoplex-AuNS systems showed the expected blue shift of the LSPR band with the irradiation time (Figure S6a) as a consequence of the pronounced reshaping of the nanostars into nanospheres observed by TEM (Figure S6b–f). Accordingly, disruption of the lipoplexes was also observed at all irradiation times (Figure S7a–d), while no modification of the lipid pattern occurred below 400 mW (Figure S7e–h).

## 4. Conclusions

The addition of plasmonic AuNSs during the preparation of lipoplexes at physiological conditions did not affect the typical lamellar pattern of such systems, since the nanoparticles were found to remain confined at the inter-lipoplexes spaces. The high electromagnetic enhancement at the tips of AuNSs by LSPR excitation with femtosecond laser pulses allowed for controlled nanoparticle heating, disrupting the structure of the lipoplexes by thermal heating and thus inducing effective pDNA release. The optimum release conditions were determined as a critical AuNS concentration of 50 pM and laser power density of 400 mW, with an efficiency that reached up to 75% at irradiation times beyond 15 min. Under these experimental conditions, the thermal heating that triggered the release of pDNA also resulted in reshaping from nanostars to nanospheres. Irradiation of the lipoplex-AuNS system did not lead to pDNA fragmentation. We have demonstrated that the femtosecond pulse laser excitation of AuNSs can be used as an external stimulus for “on-demand” pDNA release, offering an interesting strategy for potential transfection applications. Further experiments will be required to determine the viability of the present lipoplex-AuNS system and derivative systems with larger interaction specificities between lipoplex and AuNSs to control transfection assisted by femtosecond laser irradiation in different cell lines.

## Supplementary Materials

The following are available online at https://www.mdpi.com/article/10.3390/nano11061498/s1, Figure S1: Agarose gel electrophoresis experiments of GCL/DOPE-pDNA lipoplexes (without AuNSs), Figure S2: Additional UV-vis-NIR spectra, Figure S3: Additional UV-Vis-NIR spectra and TEM micrographs, Figures S4 and S5: Additional TEM micrographs, Figure S6: Additional UV-Vis-NIR spectra and TEM micrographs, Figure S7: Additional stained TEM micrographs.

## References

[1] Ponti F., Campolungo M., Melchiori C., Bono N., Candiani G. (2021). Cationic lipids for gene delivery: Many players, one goal. Chem. Phys. Lipids.

[2] Lundstrom K. (2018). Viral Vectors in Gene Therapy. Diseases.

[3] Nayak S., Herzog R.W. (2010). Progress and prospects: Immune responses to viral vectors. Gene Ther..

[4] Jones C.H., Chen C.K., Ravikrishnan A., Rane S., Pfeifer B.A. (2013). Overcoming nonviral gene delivery barriers: Perspective and future. Mol. Pharm..

[5] Donkuru M., Badea I., Wettig S., Verrall R., Elsabahy M., Foldvari M. (2010). Advancing nonviral gene delivery: Lipid- and surfactant-based nanoparticle design strategies. Nanomedicine.

[6] Ahmed T., Kamel A.O., Wettig S.D. (2016). Interactions between DNA and gemini surfactant: Impact on gene therapy: Part I. Nanomedicine.

[7] Sharma V.D., Lees J., Hoffman N.E., Brailoiu E., Madesh M., Wunder S.L., Ilies M.A. (2014). Modulation of pyridinium cationic lipid-DNA complex properties by pyridinium gemini surfactants and its impact on lipoplex transfection properties. Mol. Pharm..

[8] Zhou T., Llizo A., Li P., Wang C.X., Guo Y.Y., Ao M.Q., Bai L.L., Wang C., Yang Y.L., Xu G.Y. (2013). High transfection efficiency of homogeneous DNA nanoparticles induced by imidazolium gemini surfactant as nonviral vector. J. Phys. Chem. C.

[9] Kumar K., Barrán-Berdón A.L., Datta S., Muñoz-Úbeda M., Aicart-Ramos C., Kondaiah P., Junquera E., Bhattacharya S., Aicart E. (2015). A delocalizable cationic headgroup together with an oligo-oxyethylene spacer in gemini cationic lipids improves their biological activity as vectors of plasmid DNA. J. Mater. Chem. B.

[10] Misra S.K., Muñoz-Úbeda M., Datta S., Barrán-Berdón A.L., Aicart-Ramos C., Castro-Hartmann P., Kondaiah P., Junquera E., Bhattacharya S., Aicart E. (2013). Effects of a delocalizable cation on the headgroup of gemini lipids on the lipoplex-type nano-aggregates directly formed from plasmid DNA. Biomacromolecules.

[11] Dass C.R. (2004). Lipoplex-mediated delivery of nucleic acids: Factors affecting in vivo transfection. J. Mol. Med..

[12] Junquera E., Aicart E. (2016). Recent progress in gene therapy to deliver nucleic acids with multivalent cationic vectors. Adv. Colloid Interface Sci..

[13] Zuhorn I.S., Engberts J., Hoekstra D. (2007). Gene delivery by cationic lipid vectors: Overcoming cellular barriers. Eur. Biophys. J..

[14] Verrneulen L.M.P., Brans T., De Smedt S.C., Remaut K., Braeckmans K. (2018). Methodologies to investigate intracellular barriers for nucleic acid delivery in non-viral gene therapy. Nano Today.

[15] Gilleron J., Querbes W., Zeigerer A., Borodovsky A., Marsico G., Schubert U., Manygoats K., Seifert S., Andree C., Stoter M. (2013). Image-based analysis of lipid nanoparticle-mediated siRNA delivery, intracellular trafficking and endosomal escape. Nat. Biotechnol..

[16] Su S., Kang P.M. (2020). Recent advances in nanocarrier-assisted therapeutics delivery systems. Pharmaceutics.

[17] Hashimoto T., Hirata T., Yuba E., Harada A., Kono K. (2020). Light-activatable transfection system using hybrid vectors composed of thermosensitive dendron lipids and gold nanorods. Pharmaceutics.

[18] Huang X.H., Jain P.K., El-Sayed I.H., El-Sayed M.A. (2008). Plasmonic photothermal therapy (PPTT) using gold nanoparticles. Lasers Med. Sci..

[19] Singhana B., Slattery P., Chen A., Wallace M., Melancon M.P. (2014). Light-activatable gold nanoshells for drug delivery applications. AAPS PharmSciTech.

[20] Perez-Hernandez M., del Pino P., Mitchell S.G., Moros M., Stepien G., Pelaz B., Parak W.J., Galvez E.M., Pardo J., de la Fuente J.M. (2015). Dissecting the molecular mechanism of apoptosis during photothermal therapy using gold nanoprisms. ACS Nano.

[21] Guerrero-Martinez A., Barbosa S., Pastoriza-Santos I., Liz-Marzan L.M. (2011). Nanostars shine bright for you Colloidal synthesis, properties and applications of branched metallic nanoparticles. Curr. Opin. Colloid Interface Sci..

[22] Espinosa A., Silva A.K.A., Sanchez-Iglesias A., Grzelczak M., Pechoux C., Desboeufs K., Liz-Marzan L.M., Wilhelm C. (2016). Cancer cell internalization of gold nanostars impacts their photothermal efficiency in vitro and in vivo: Toward a plasmonic thermal fingerprint in tumoral environment. Adv. Healthc. Mater..

[23] Weissleder R. (2001). A clearer vision for in vivo imaging. Nat. Biotechnol..

[24] Smith A.M., Mancini M.C., Nie S.M. (2009). Second window for in vivo imaging. Nat. Nanotechnol..

[25] Tsai M.F., Chang S.H.G., Cheng F.Y., Shanmugam V., Cheng Y.S., Su C.H., Yeh C.S. (2013). Au nanorod design as light-absorber in the first and second biological near-infrared windows for in vivo photothermal therapy. ACS Nano.

[26] Ahijado-Guzman R., Sanchez-Arribas N., Martinez-Negro M., Gonzalez-Rubio G., Santiago-Varela M., Pardo M., Pineiro A., Lopez-Montero I., Junquera E., Guerrero-Martinez A. (2020). Intercellular trafficking of gold nanostars in uveal melanoma cells for plasmonic photothermal therapy. Nanomaterials.

[27] Gonzalez-Rubio G., Guerrero-Martinez A., Liz-Marzan L.M. (2016). Reshaping, fragmentation, and assembly of gold nanoparticles assisted by pulse lasers. Acc. Chem. Res..

[28] Yuan H.K., Khoury C.G., Hwang H., Wilson C.M., Grant G.A., Vo-Dinh T. (2012). Gold nanostars: Surfactant-free synthesis, 3D modelling, and two-photon photoluminescence imaging. Nanotechnology.

[29] Muñoz-Úbeda M., Misra S.K., Barrán-Berdón A.L., Datta S., Aicart-Ramos C., Castro-Hartmann P., Kondaiah P., Junquera E., Bhattacharya S., Aicart E. (2012). How does the spacer length of cationic gemini lipids influence the lipoplex formation with plasmid DNA? Physicochemical and biochemical characterizations and their relevance in gene therapy. Biomacromolecules.

[30] Muñoz-Úbeda M., Misra S.K., Barrán-Berdón A.L., Aicart-Ramos C., Sierra M.B., Biswas J., Kondaiah P., Junquera E., Bhattacharya S., Aicart E. (2011). Why is less cationic lipid required to prepare lipoplexes from plasmid DNA than linear DNA in gene therapy?. J. Am. Chem. Soc..

[31] Pelaz B., del Pino P., Maffre P., Hartmann R., Gallego M., Rivera-Fernandez S., de la Fuente J.M., Nienhaus G.U., Parak W.J. (2015). Surface functionalization of nanoparticles with polyethylene glycol: Effects on protein adsorption and cellular uptake. ACS Nano.

[32] Ahijado-Guzman R., Gonzalez-Rubio G., Izquierdo J.G., Banares L., Lopez-Montero I., Calzado-Martin A., Calleja M., Tardajos G., Guerrero-Martinez A. (2016). Intracellular pH-induced tip-to-tip assembly of gold nanorods for enhanced plasmonic photothermal therapy. ACS Omega.

