# Supplementary material for: Controlled pDNA Release in Gemini Cationic Lipoplexes by Femtosecond Laser Irradiation of Gold Nanostars
Source: Nanomaterials (Basel). 2021 Jun 5;11(6):1498. doi: 10.3390/nano11061498 (PMC8229200; doi:10.3390/nano11061498)
Supplement: Supplementary file 1 [file nanomaterials-11-01498-s001.zip › nanomaterials-1217635-supplementary.pdf]

Supplementary Materials

Controlled pDNA Release in Gemini Cationic Lipoplexes by Femtosecond Laser Irradiation of Gold Nanostars

Natalia Sánchez-Arribas [1], Pablo Díaz-Núñez [2], José Osío Barcina [3], Emilio Aicart [1], Elena Junquera [1,*] and Andrés Guerrero-Martínez [1,*]

[1] Departamento de Química Física, Facultad de Ciencias Químicas, Universidad Complutense de Madrid, 28040 Madrid, Spain; natsanch@ucm.es (N.S.-A.); aicart@quim.ucm.es (E.A.)
[2] Instituto de Fusión Nuclear, "Guillermo Velarde", Universidad Politécnica de Madrid, José Gutiérrez Abascal 2, E-28006 Madrid, Spain; pablodn87@gmail.com
[3] Departamento de Química Orgánica, Facultad de Ciencias Químicas, Universidad Complutense de Madrid, 28040 Madrid, Spain; josio@quim.ucm.es
* Correspondence: junquera@quim.ucm.es (E.J.); aguerrero@quim.ucm.es (A.G.-M.)

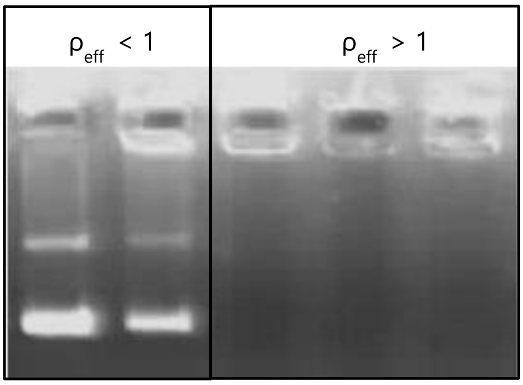

Figure S1. Agarose gel electrophoresis experiments of IGCL/DOPE-pDNA lipoplexes (without AuNSs). Adapted from *Biomacromolecules* **2013**, 14, 3951–3963 with permission of ACS Publications.

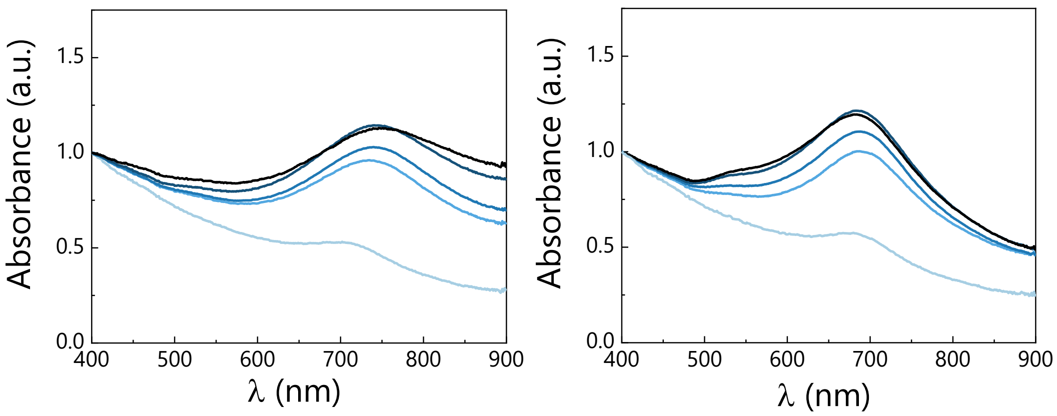

(a) **(b)**

Figure S2. Normalized UV-vis-NIR spectra of the lipoplex-AuNS system under irradiated conditions, at 400 mW (a) and 700 mW (b) for 5 min, and at increasing AuNS concentration 20, 50, 100, 150 and 200 pM (from light to dark color).

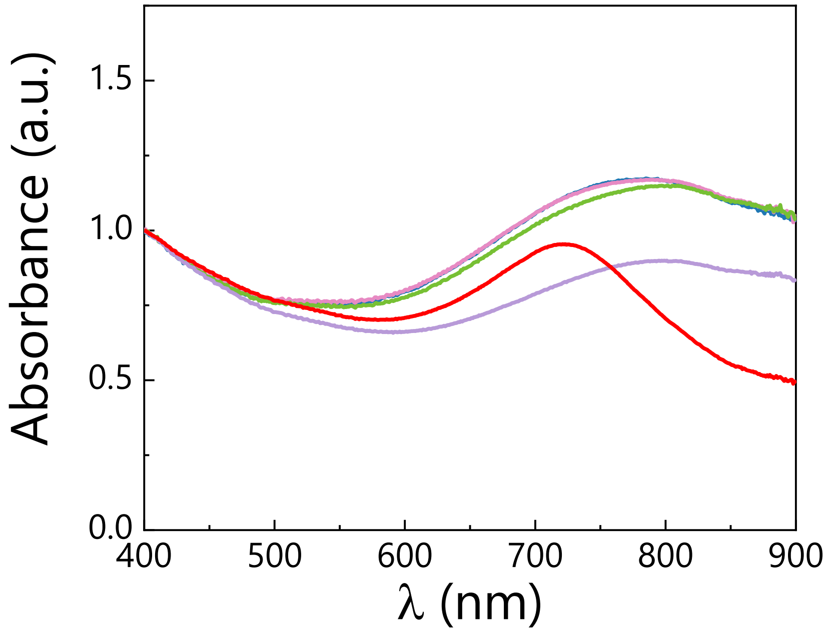

(a)

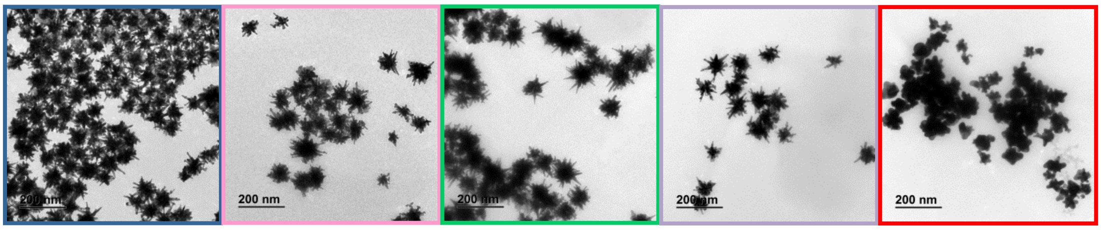

(b) **(c)** **(d)** **(e)** **(f)**

Figure S3. (a) Normalized UV-vis-NIR spectra of the lipoplex-AuNS system (100 pM AuNSs) under non-irradiated (blue line) and irradiated conditions, at 50 mW (pink line), 100 mW (green line), 200 mW (purple line) and 400 mW (red line) for 5 min. TEM micrographs of the lipoplex-Au NS systems (100 pM AuNSs) under non-irradiated conditions **(b)** and irradiated conditions at 50 mW **(c)**, 100 mW **(d)**, 200 mW **(e)**, and 400 mW **(f)** for 5 min.

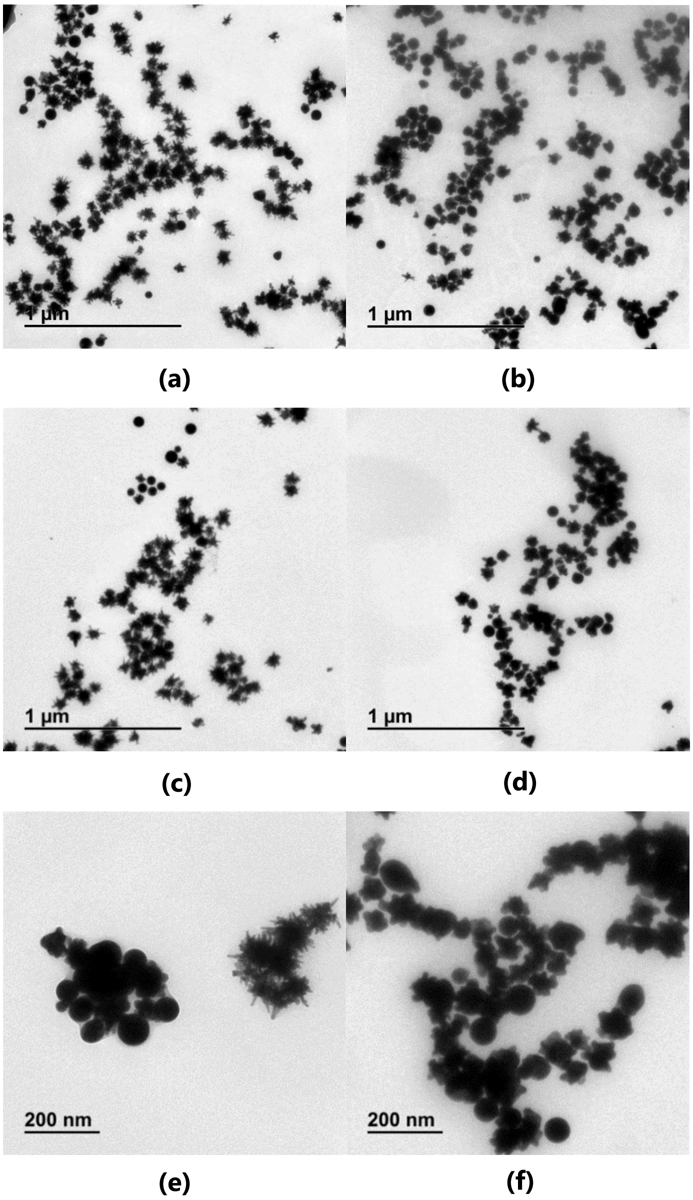

Figure S4. TEM micrographs of the lipoplex-AuNS system (200 pM AuNSs) at irradiated conditions of 400 mW (a,c,e) and 700 mW (b,d,f) for 5 min.

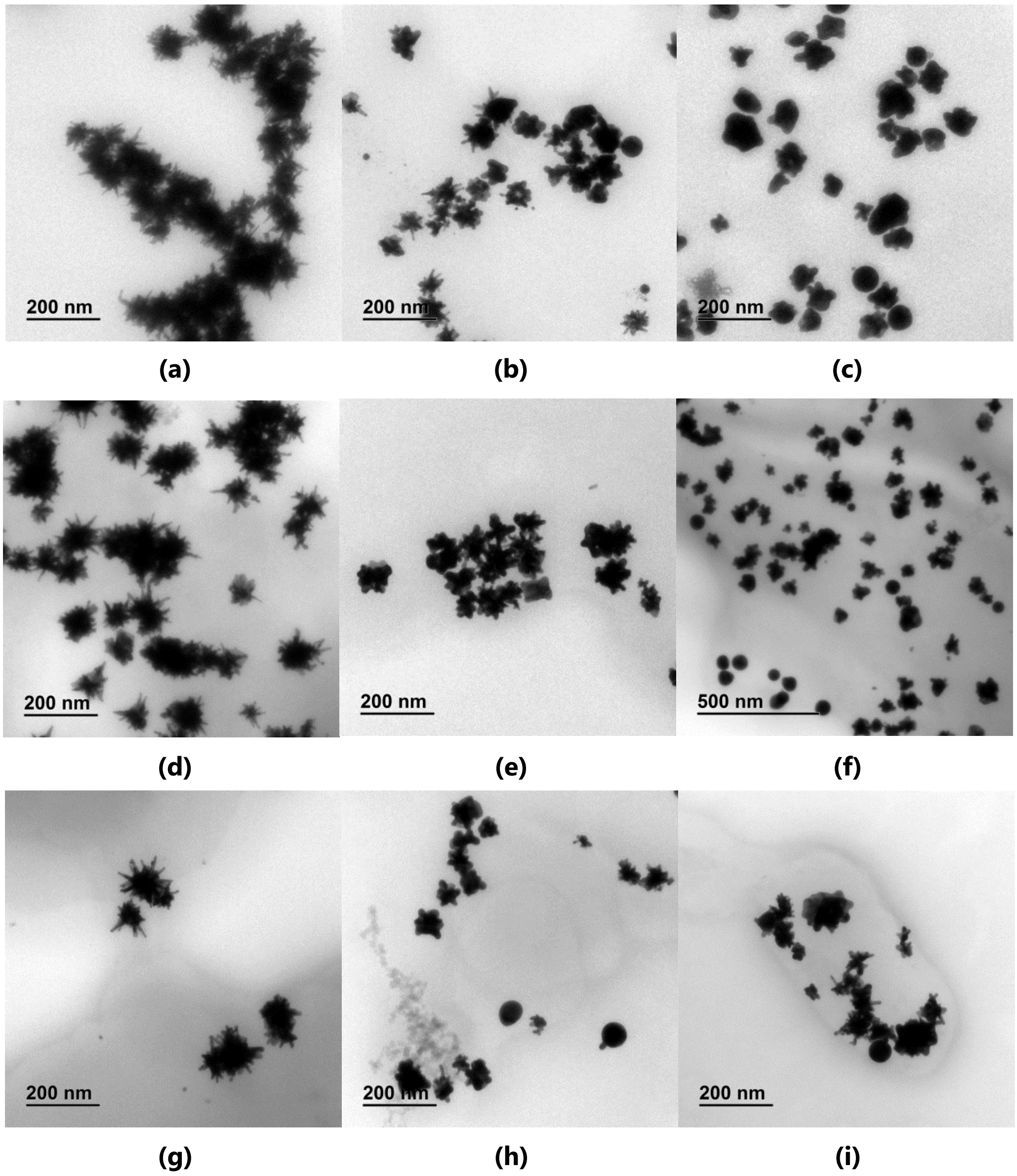

Figure S5. TEM micrographs of the lipoplex-Au NS system (150 pM AuNSs: a-c; 50 pM AuNSs: d-f; 20 pM AuNSs: g-i) at non-irradiated conditions (a,d,g) and at irradiated conditions of 400 mW (b,e,h) and 700 mW (c,f,i) for 5 min.

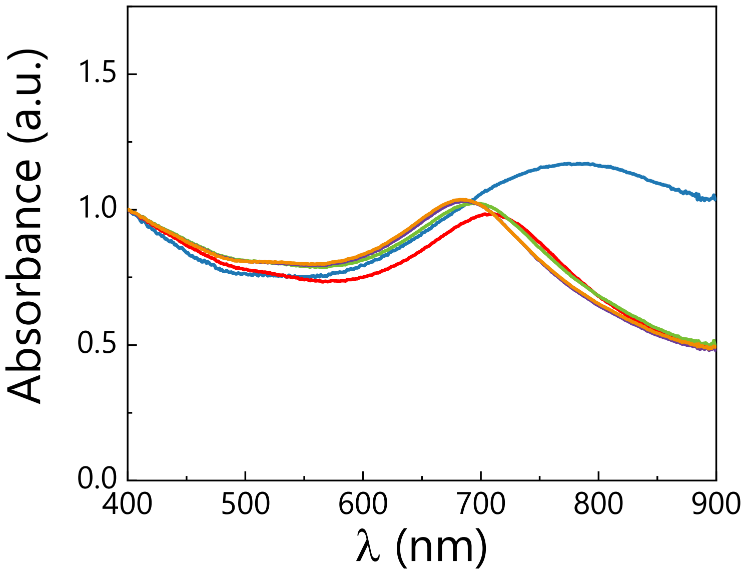

(a)

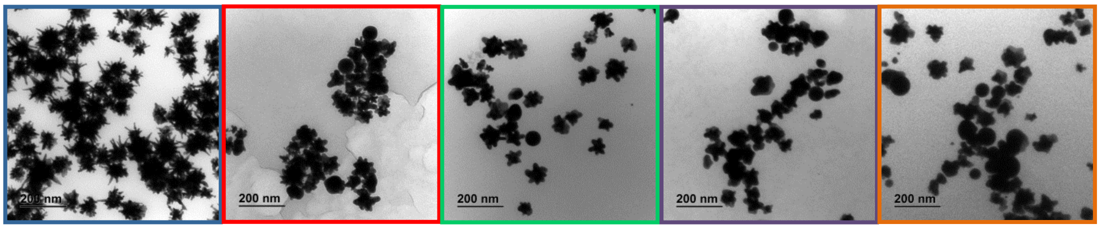

(b) **(c)** **(d)** **(e)** **(f)**

Figure S6. **(a)** Normalized UV-vis-NIR spectra of the lipoplex-AuNS system (100 pM AuNSs) under non-irradiated (blue line) and irradiated conditions, at 400 mW and different times: 5 min (red line), 15 min (green line), 30 min (purple line) and 60 min (orange line). TEM micrographs of the lipoplex-AuNS system (100 pM AuNSs) under non-irradiated **(b)** and irradiated conditions at 400 mW and different times: 5 min **(c)**, 15 min **(d)**, 30 min **(e)**, and 60 min **(f)**.

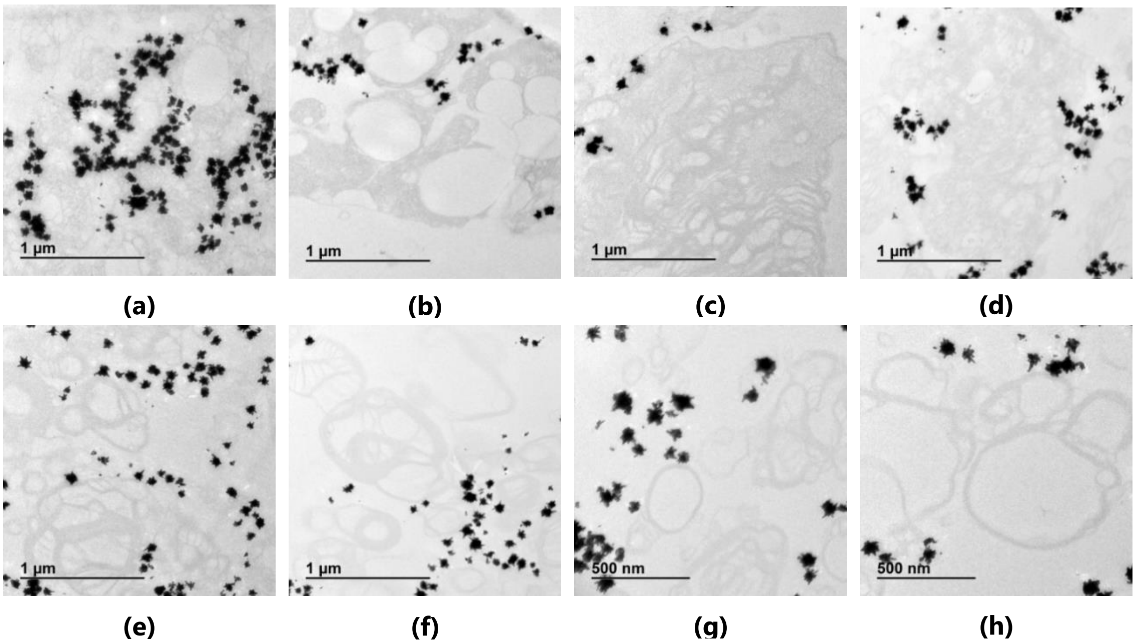

Figure S7. Stained TEM micrographs of the lipoplex-AuNSs (100 pM AuNSs) under irradiated conditions at 400 mW (a-d) and 100 mW (e-h) for 5 min (a,e), 15 min (b,f), 30 min (c,g) and 60 min (d,h).
